# First Morphological and Molecular Characterization of *Paratylenchus vandenbrandei* (Rhabditida: Tylenchulidae) in Iran

**DOI:** 10.2478/jofnem-2023-0058

**Published:** 2023-12-14

**Authors:** Abbas Abdolkhani, Sedighe Azimi

**Affiliations:** Department of Plant Protection, Faculty of Agriculture, Shahid Chamran University of Ahvaz, Ahvaz, Iran

**Keywords:** D2-D3 expansion segments of LSU rDNA, ITS rDNA, Khuzestan province, morphology, phylogeny

## Abstract

*Paratylenchus vandenbrandei*, has been recovered from the rhizospheric soil of Euphrates poplar (*Populus euphratica*) in the Karkheh protected area of Khuzestan province, southwestern Iran. The species was identified as *P. vandenbrandei* by the presence of three lines in the lateral fields; conoid rounded lip region; presence of submedian lobes, a stylet 24.0–28.8 μm long; an excretory pore at the level of the anterior part of the pharyngeal bulb; a round-to-oval spermatheca; presence of vulval flaps; and a conoid tail, with a terminus that is rounded or slightly pointed in some specimens. Males have a conoid tail, with a rounded-to-slightly-pointed terminus. The phylogenetic relationships of the species were reconstructed and investigated using partial sequencing of the D2-D3 expansion segments of large subunits, as well as internal transcribed spacer regions (LSU D2-D3 and ITS rDNA) based on Bayesian inference (BI). *P. vandenbrandei* has formed a clade with *P. neonanus*, *P. minor*, *P. nainianus*, *P. chongqinjensis*, *P. pedrami*, *P. baldaccii*, *P. leptos* and *P. rostrocaudatus* with maximal support (BPP = 1.00). To the best of our knowledge, this is the first report of *P. vandenbrandei* in Iran and the first molecular characterization of the species worldwide.

The plant-parasitic nematodes of the genus *Paratylenchus* Micoletzky, 1922 are obligate ectoparasites of a large variety of plants. They are commonly known as pin nematodes and are common in orchard soils (Siddiqi, 2000). They exist worldwide and have a remarkable adaptability to varying environmental conditions such as hot or cold temperatures and drought (Clavero-Camacho et al., 2021a; Palomares-Rius et al., 2022; Rosmaninho et al., 2022).

Species identification in the genus *Paratylenchus* is a very problematic task because of member species’ high morphological and morphometric similarity and high levels of intra-specific variability, as well as the large number of species. Thus, molecular data are required to separate closely related species (Van den Berg et al., 2014; Clavero-Camacho et al., 2021a; Palomares-Rius et al., 2022). Additionally, morphological characters that are usually used for species identification can be influenced by environmental and other external factors, such as temperature, host, and population size (Clavero-Camacho et al., 2021a). The D2-D3 regions of the 28S rRNA gene and ITS1 have proved more effective in species identification compared to partial 18S, as both these molecular markers display more species variability than partial 18S (Rosmaninho et al., 2022). In addition to the 28S rRNA gene and ITS rRNA gene, the COI mtDNA gene is a beneficial tool for accurate identification of *Paratylenchus* spp. and the detection of the cryptic diversity in pin nematodes (Clavero-Camacho et al., 2021a, b; Singh et al., 2021; Palomares-Rius et al., 2022; Rosmaninho et al., 2022; Álvarez-Ortega et al., 2023). With the increasing use of molecular markers for species identification in the genus in recent years, the number of known cryptic species within *Paratylenchus* is likely to increase (Clavero-Camacho et al., 2021b; Palomares-Rius et al., 2022).

Thirty-seven species of the genus *Paratylenchus* have been reported from various plants in Iran (Ghaderi et al., 2019; Hosseinvand et al., 2020). However, most of these studies did not use molecular methods for their identification. The present research aims to conduct an identification with morphological and morphometric data for the Iranian population of *Paratylenchus vandenbrandei* De Grisse, 1962. Additionally, it provides molecular characterization of the detected species using D2-D3 expansion segments of 28S rRNA gene and internal transcribed spacer regions (ITS rRNA) and compares the phylogenetic relationships of the species with available sequences from other pin nematode species deposited in Genbank.

## Materials and Methods

### Nematode extraction and morphological observations

Several soil samples were collected from the rhizosphere of Euphrates poplar (*Populus euphratica* Oliv.) in Karkheh protected area of Khuzestan province, southwestern Iran. The centrifugal-flotation technique (Jenkins, 1964) and the tray method (Whitehead and Hemming, 1965) were used for extracting the nematodes from soil samples.

The collected specimens were killed in a hot 4% formaldehyde solution and transferred to anhydrous glycerin according to the De Grisse method (1969). Observations and measurements were conducted using a Leitz SM-LUX light microscope (Leitz Corporation, Wetzlar, Germany) equipped with a drawing tube. Some of the specimens were photographed using an Olympus DP72 digital camera attached to an Olympus BX51 light microscope (Olympus Corporation, Tokyo, Japan).

### DNA extraction, PCR and sequencing

For molecular analyses, single female specimens were picked out, examined in a drop of distilled water on a temporary slide under the light microscope, transferred to 5 μl of TE buffer (10 mM Tris-Cl, 0.5 mM EDTA; pH 9.0) on a clean slide, and then crushed using a cover slip. Each suspension was collected by adding 10 μl TE buffer. The DNA samples were stored at −20 °C until they could be used as PCR templates. Primers for LSU rDNA D2-D3 amplification were forward primer D2A (5′-ACAAGTACCGTGAGGGAAAGT-3′) and reverse primer D3B (5′-TCGGAAGGAACCAGCTACTA-3′) (Nunn, 1992). Primers for amplification of ITS rDNA were forward primer rDNA1 (5′-TTGATTACGTCCCTGCCCTTT-3′) and reverse primer rDNA1.58S (5′-ACGAGCCGAGTGATCCACCG-3′) (Subbotin et al., 2000). To amplify the above-mentioned segments of DNA, the polymerase chain reactions (PCRs) were performed as previously described (Jumaah & Azimi 2022). Amplification success was evaluated using electrophoresis on 1% agarose gel. The PCR products were then subjected to sequencing using an Applied Biosystems 3500 (ABI) sequencer (Pishgam Corporation, Tehran, Iran). The newly obtained sequences were deposited into the GenBank database (accession numbers OR345519 and OR345520 for LSU D2-D3, and OR354721 and OR354722 for ITS rDNA).

### Phylogenetic analyses

The newly obtained sequences of the D2-D3 fragments of LSU rDNA and ITS rDNA and additional sequences of relevant species were selected after a nucleotide basic local alignment search tool (BLASTn) search. The sequences were aligned by Clustal X version 2 using the default parameters (Larkin et al., 2007). The outgroup taxa were chosen according to previous studies (Clavero-Camacho et al., 2021a; Singh et al., 2021). The editing of both alignments was performed manually in the MEGA7 program (Kumar et al., 2016). Based on the Akaike information criterion, the base substitution model was selected using MrModeltest2 (Nylander, 2004). A general time-reversible model, including among-site rate heterogeneity and estimates of invariant sites (GTR + G + I), was used in both phylogenies. A Bayesian analysis using MrBayes v3.1.2 (Ronquist and Huelsenbeck, 2003) was performed to infer the phylogenetic trees, running the chains for four million generations. After discarding burn-in samples and evaluating convergence, the remaining samples were retained for further analyses. The Markov Chain Monte Carlo (MCMC) method, within a Bayesian framework, was used to determine equilibrium distribution and help estimate the posterior probabilities of the phylogenetic trees (Larget and Simon, 1999) using the 50% majority rule. Bayesian posterior probability (BPP) values higher than 0.50 were given to appropriate clades. The output files of the phylogenetic program were visualized using Dendroscope v3.2.8 (Huson and Scornavacca, 2012) and digitally drawn in CorelDRAW software version 23 (Corel Corporation, Ottawa, Canada).

## Results

**Figure 1:**
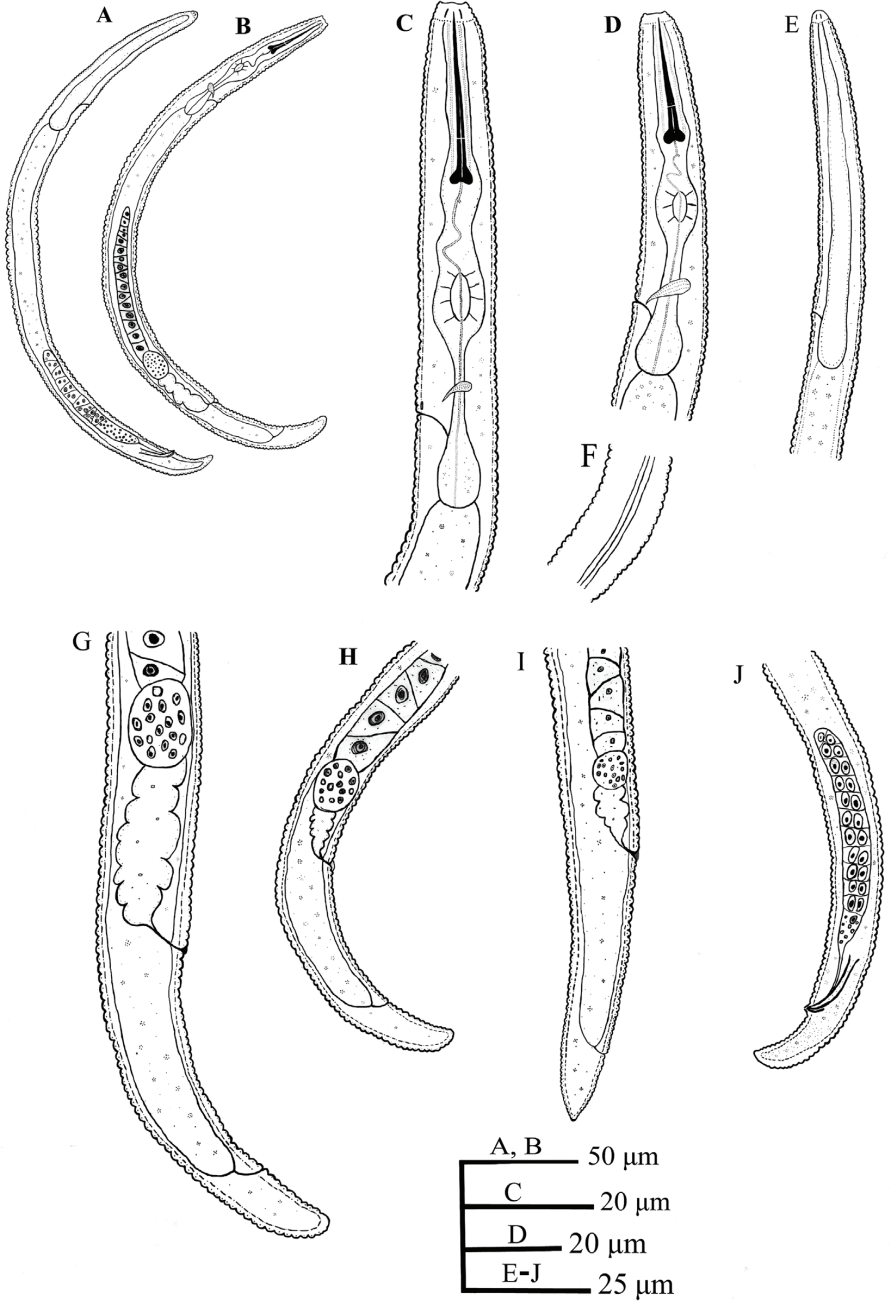
Line drawings of *Paratylenchus vandenbrandei* De Grisse, 1962. A, E, J: Male. A: Entire body; E: Anterior body region; J: Posterior body region; B-D, F-I: Female. B: Entire body; C, D: Anterior body region; F: Lateral field at mid-body; G-I: Posterior body region.

**Figure 2:**
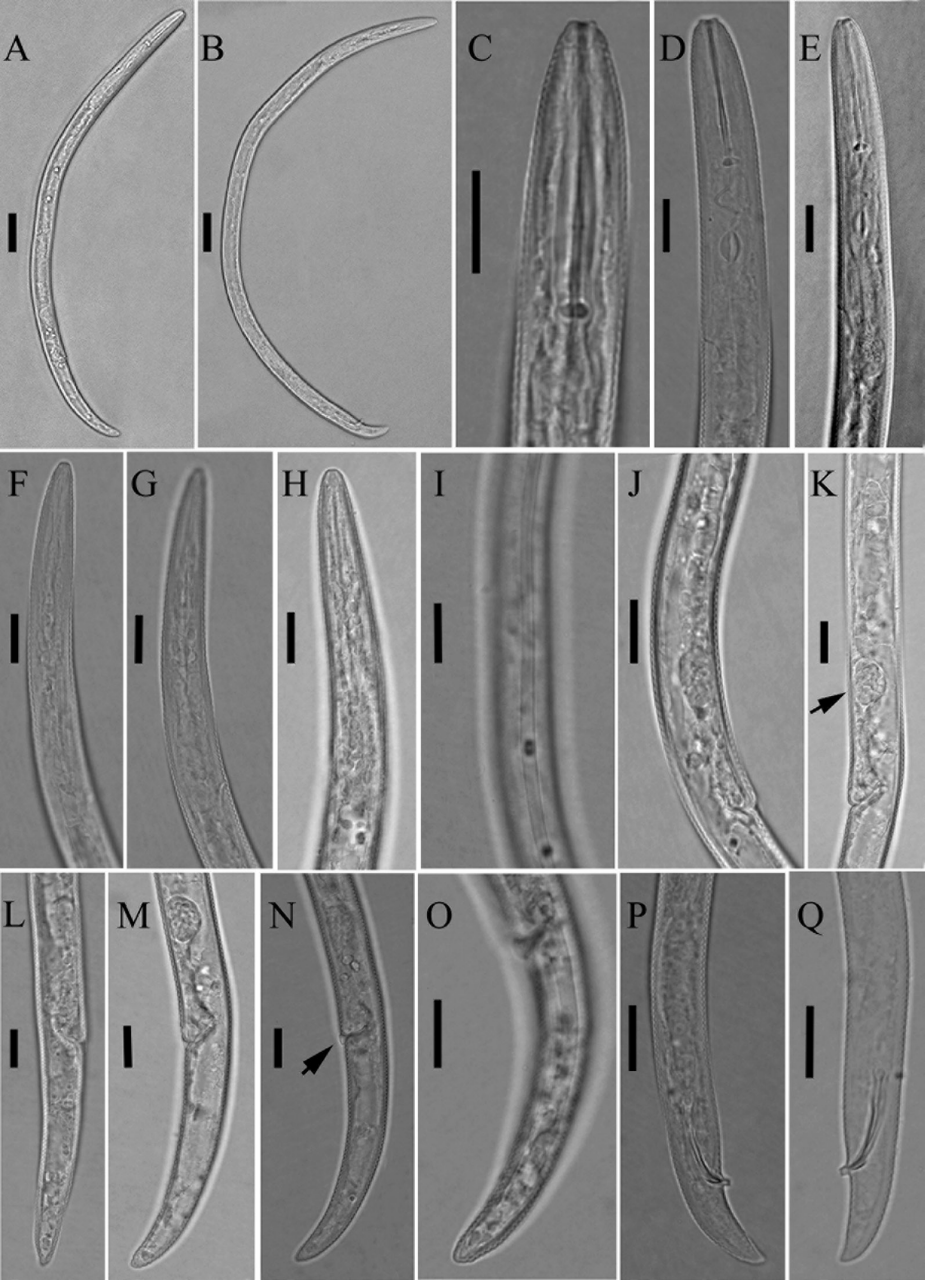
Light photomicrographs of *Paratylenchus vandenbrandei* De Grisse, 1962. A, C-E, I-O: Female. A: Entire body; C-E: Anterior body region; I: Lateral field at mid-body; J, K: Reproductive system (the arrow indicates the spermatheca); L-O: Posterior body region (the arrow indicates the advulval flap); B, F-H, P, Q: Male. B: Entire body; F-H: Anterior body region; P, Q: Posterior body region. (Scale bars: A, B = 20 μm; C-Q = 10 μm).

**Figure 3:**
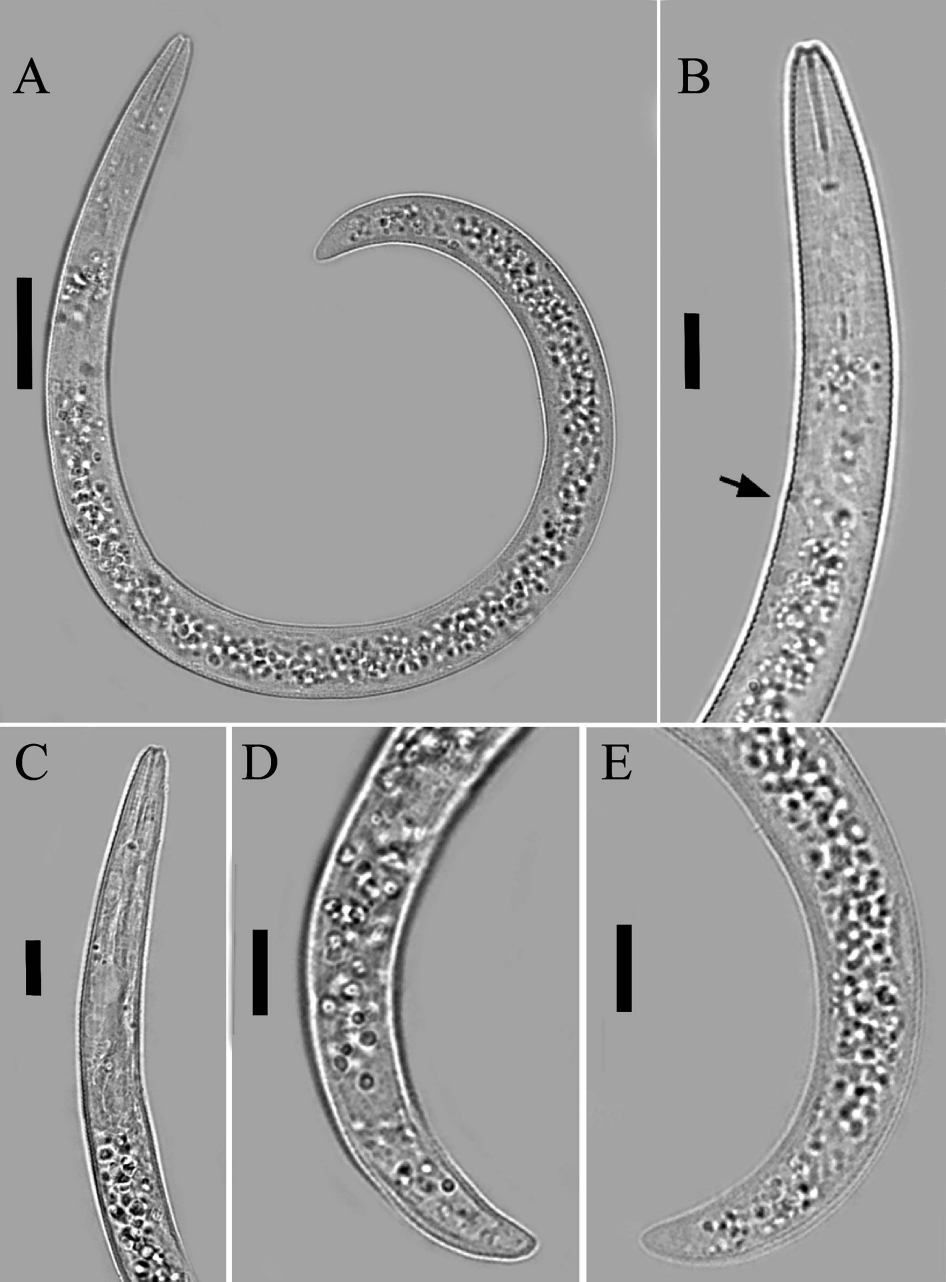
Light photomicrographs of *Paratylenchus vandenbrandei* De Grisse, 1962. Juvenile. A: Entire body; B, C: Anterior body region (the arrow indicates the excretory pore); D, E: Posterior body region. (Scale bars: A = 20 μm; B-E = 10 μm).

(Figures 1–3; Table 1).

**Table 1.** Morphometrics of *Paratylenchus vandenbrandei* from Khuzestan province, Iran, and comparison with the original description. All measurements are in μm and in the form: mean ± s.d. (range).

**Character**	**Present study**		**De Grisse, 1962**	
	
	**Female**	**Male**	**Female**	**Male**
n	11	3	19	3
L	296 ±12 (259–314)	308 ± 53 (275–369)	269.0 ± 3.6 (248–290)	261 (248–269)
a	22.4 ± 0.8 (21.3–23.2)	29.6 ± 3.3 (27.2–32.0)	20 (14–24)	28 (27–30)
b	3.9 ± 0.2 (3.7–4.5)	4.4 ± 0.6 (4.2–4.6)	3.5 (3.2–3.7)	-
c	20.0 ± 2.3 (15–22)	19.6 ± 1.9 (18.3–21.0)	18 (15–21)	16.6 (16.5–16.8)
c′	2.0 ± 0.2 (1.6–2.3)	1.7 ± 0.07 (1.7–1.8)	2–3	-
V	80.0 ± 2.1 (79.0–84.5)	-	82 (81–86)	-
T	-	19.2 ± 2.7 (17.3–21.2)	-	-
Stylet length	27 ± 1.7 (24.0–28.8)	-	31.0 ± 5.6 (28–33)	-
Conus length	17.1 ± 2.5 (14.7–19.5)	-	-	-
m	71.1 ± 1.5 (65.1–74.2)	-	-	-
DGO	3.5 ± 0.5 (3.2–4.2)	-		-
Anterior end to excretory pore	63.4 ± 3.0 (58–67)	61.0 ± 8.4 (55–67)	59–68	58–64
Pharynx length	74.6 ± 5.9 (68.5–81.2)	72.0 ± 9.8 (65–79)	-	-
Maximum body width	12.9 ± 0.8 (12.0–14.0)	10.9 ± 3.1 (10.1–12.5)	-	-
Vulva body width	12.2 ± 1.0 (11.5–13.5)	-	-	-
Anal body width	7.5 ± 2.0 (6.5–9.2)	9.1 ± 0.8 (8.5–9.7)	-	-
Head-vulva	239.5 ± 8.5 (228–248)	-	-	-
Head-anus	275.2 ±10.1 (264–292)	-	-	-
Vulva-anus	39.5 ± 6.5 (29–48)	-	-	-
St/L%	11.4 ± 0.7 (9.5–12.3)	-	-	-
Tail length	15.1 ± 1.4 (14.0–17.5)	16.1 ± 1.5 (15.0–17.2)	15 (12–18)	15.6 (15–16)
Spicule length	-	16.2 ± 1.7 (15.5–17.5)	-	14.5 (14–15)
Gubernaculum length	-	3.5 ± 1.7 (3.0–4.0)	-	2.5–3.0

### Iranian population of *Paratylenchus vandenbrandei*

#### Female

Body slender, arcuate ventrally to open C-shaped after heat fixation. Cuticle is finely annulated, 0.6–0.9 μm wide at mid-body. Lateral field has three incisures, occupying 25–35% of the body diameter, with non-areolated bands. Lip region is conoid rounded, slightly offset from the body, 2.7–3.5 μm high and 5.2–6.5 μm wide, with submedian lobes present and cephalic framework weak. Stylet is straight and robust, with rounded basal knobs, slightly posteriorly directed, 3.1–3.5 μm across. The pharyngeal region is of the typical paratylenchoid type. The median pharyngeal bulb is slender and elongate, having distinct large valves; the isthmus is short, slender, and surrounded by a nerve ring; the basal bulb is pyriform and offset from the intestine. The excretory pore is located at the level of the anterior part of the pharyngeal bulb, posterior to the hemizonid. The reproductive system is monodelphic-prodelphic, composed of an outstretched ovary; well-developed spermatheca that are round to oval and filled with rounded sperm; and vulva a transverse slit, with vulval flaps present, and post-vulval uterine sac absent. The tail is conoid and finely annulated, gradually tapering, with a rounded terminus that can be slightly pointed in some specimens.

#### Male

Body slender and arcuate ventrally to open C-shaped after heat fixation. The cuticle is finely annulated. The lip region is narrower than that of the female, slightly truncated, and continuous with the body, with submedian lobes indistinct, and cephalic framework weak. The stylet is absent, the pharynx non-functional, and the procorpus, metacorpus, and basal bulb inconspicuous. Testis are outstretched, with small spermatozoa. The spicule is slender, slightly curved towards the end. The gubernaculum is curved. The bursa is absent. The tail is conoid, tapering gradually, with a rounded-to-slightly-pointed terminus.

#### Juvenile

Juveniles were similar in morphology to adult females. The lip region has submedian lobes, stylet length 19.4 (17.6–22.0) μm; the pharynx is underdeveloped; and the anus is indistinct in most specimens. The posterior body has a rounded terminus.

#### Relationships

According to species grouping by Ghaderi et al. (2014, 2016), *P. vandenbrandei* belongs to group two. By having three lines in the lateral fields, stylet length less than 40 μm and with advulval flaps present, it appears most similar to *P. aquaticus* Merny, 1966, *P. humilis* Raski, 1975a and *P. perminimus* Siddiqi, 1996. It differs from *P. aquaticus* due to its longer stylet length (24.0–28.8 *vs* 16.0–20.0 μm), shorter DGO (3.2–4.2 *vs* 5–6), lower *a* ratio (21.3–23.2 *vs* 26.0–39.0), shorter tail (14–17.3 *vs* 20.0–24.0 μm), lower ć ratio (1.6–2.3 *vs* 3.7), absent post-vulval uterine sac (*vs* present), shorter spicules (15.5–17.5 *vs* 21.0–22.0 μm) and absent bursa (*vs* present).

It is distinguished from *P. humilis* by its longer body (259–314 *vs* 170–190 μm), higher b ratio (3.7–4.5 *vs* 3.2–3.5), longer stylet (24.0–28.8 *vs* 16–19 μm), greater distance of anterior end to excretory pore (58–67 *vs* 43–50 μm), tail tip rounded or slightly pointed in some specimens (*vs* a slight digitate appearance with a rounded to almost acute terminus) and longer spicules (15.5–17.5 *vs* 12–14 μm). It differs from *P. perminimus* with its longer body (259–314 *vs* 160–200 μm), longer stylet (24–28 *vs* 20–22 μm), greater distance from the anterior end to the excretory pore (58–67 *vs* 43–51 μm), slightly longer tail (14.0–17.5 *vs* 10–14 μm), longer spicules (15.5–17.5 *vs* 11.5 μm) and tail terminus in the male (with a rounded to slightly pointed terminus *vs* rounded terminus). It also differs from *P. neonanus* Mathur, Khan & Prasad, 1967, and *P. nainianus* Edward & Misra, 1963, two species with close phylogenetic affinities, in that it has three lines in the lateral fields (*vs* four lines).

#### Remarks

Compared with the type population from Kenya by De Grisse, 1962, the stylet is slightly shorter (24.0–28.8 *vs* 28–33 μm), and the spicules are longer (15.5–17.5 *vs* 14–15 μm). Compared with the population from Srilanka reported by Raski, 1975b, stylet is shorter (24.0–28.8 *vs* 32–36 μm). Compared with the Spanish population reported by Gomez-Barcina et al., 1990, both the spicules and gubernaculum are shorter (15.5–17.5 *vs* 23 μm and 3.0–4.0 *vs* 4.7 μm, respectively, for only one male). Additionally, the a ratio is higher (21.3–23.2 *vs* 14–20) compared to the population from Guadeloupe described by Van den Berg and Quénéhervé (1999).

The presently studied species was recovered from the rhizospheric soil of Euphrates poplar collected from the Karkheh protected area in Khuzestan province, southwest Iran. The GPS information of the sampling site is 31°56″07.8″N, 48°15′47.4″E. *P. vandenbrandei* is herein reported for the first time in Iran.

### Molecular characterization and phylogenetic relationships

#### D2-D3 fragments of 28S rDNA phylogeny

To reconstruct the 28S rDNA tree, newly obtained 696 and 650 nt long partial sequences of the D2-D3 region with accession numbers OR345519 and OR345520 were used. The BLAST search using these sequences revealed they have 91.60% and 93.64% identity with the LSU sequence of *Paratylenchus neonanus* (ON873227). Sequence variation between *P. vandenbrandei* and *P. neonanus* was 51–53 nucleotides and seven gaps (0.10%); sequence variation between *P. vandenbrandei* and *P. nainianus* was 58 nucleotides and three gaps in the same region (0.12%).

**Figure 4:**
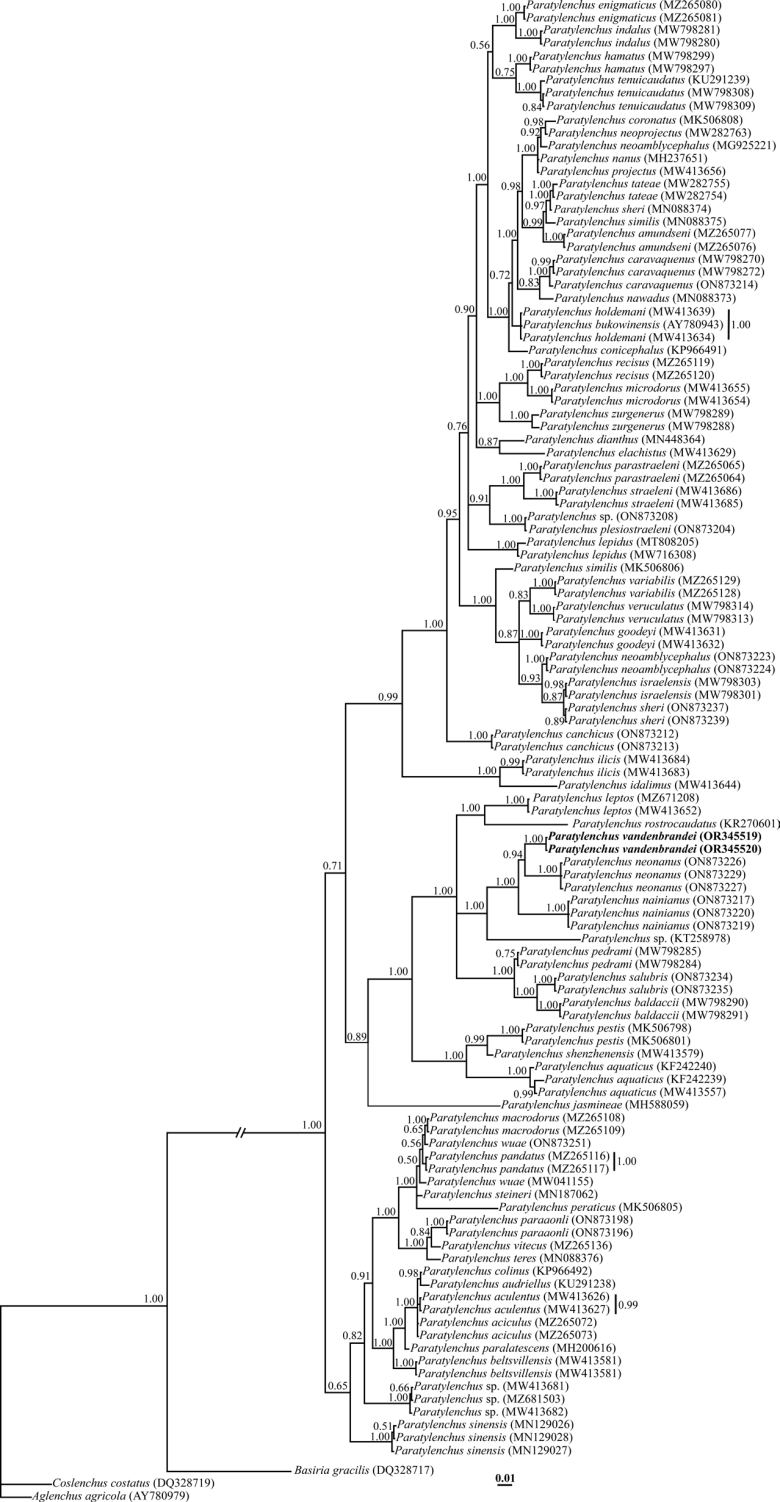
Bayesian 50% majority rule consensus tree inferred from analysis of the D2-D3 domains of the LSU rDNA sequences of *Paratylenchus vandenbrandei* De Grisse, 1962. under the GTR + G + I model. Bayesian posterior probability values of more than 0.50 are given for appropriate clades. New sequences are indicated in bold.

Three sequences from the family Tylenchidae Örley, 1880 (DQ328717, DQ328719 and AY780979) were used as outgroups for a total of 114 sequences from the *Paratylenchus* species. This *dataset comprised* 799 total characters. The phylogenetic tree inferred using this dataset is presented in Figure 4. In this tree, *P. vandenbrandei* forms a clade with *P. neonanus* and *P. nainianus* with maximal support (BPP = 1.00).

#### Partial ITS rDNA phylogeny

Two identically aligned sequences of ITS rDNA (OR354721, OR354722) were generated for the new species. A BLAST search using these sequences revealed that they have 86.92% identity with *P. nainianus* (ON873186). Sequence variation between *P. vandenbrandei* and *P. nainianus* was 40 nucleotides and eight gaps (0.14%); its variation compared to *P. minor* was 64 nucleotides and 28 gaps (0.34%); with *P. chongqinjensis* Li, Wang, Xie & Xu, 2019, it was 76 nucleotides and 16 gaps (0.30%); with *P. pedrami* Clavero-Camacho, Cantalapiedra-Navarrete, Archidona-Yuste, Castillo & Palomares-Rius, 2021, it was 55 nucleotides and 12 gaps (0.23%); with *P. baldaccii,* it was 66 nucleotides and 16 gaps (0.24%); and with *P. leptos*, it was 73 nucleotides and 21 gaps (0.25%); and with *P. rostrocaudatus*, it was 78 nucleotides and 20 gaps (0.28%).

**Figure 5:**
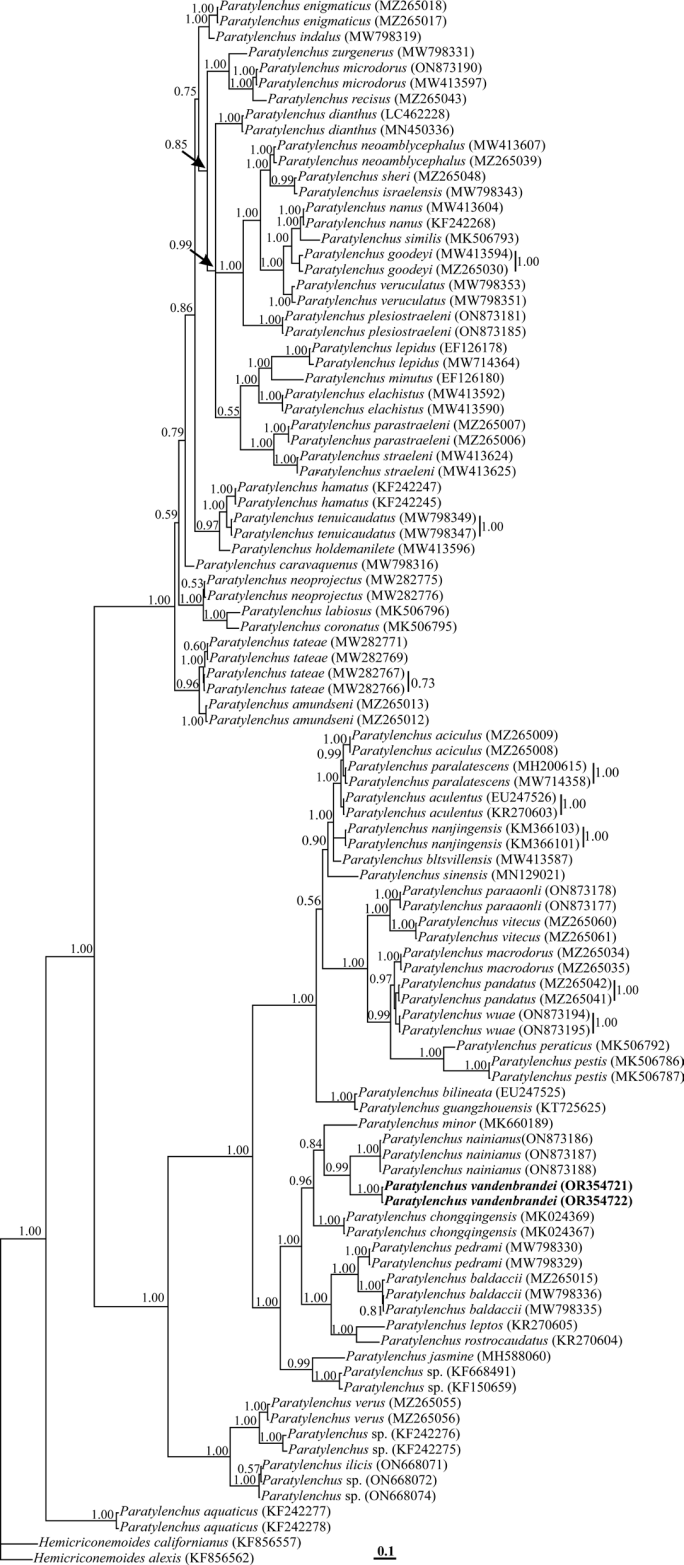
Bayesian 50% majority rule consensus tree inferred from analysis of the ITS rDNA of *Paratylenchus vandenbrandei* De Grisse, 1962. under the GTR + G + I model. Bayesian posterior probability values of more than 0.50 are given for appropriate clades. New sequences are indicated in bold.

Two sequences of the genus *Hemicriconemoides* Chitwood & Birchfield, 1957 (KF856557, KF856562) were used as outgroups for a total of 99 sequences of the *Paratylenchus* species. This dataset comprised 1101 total characters. The phylogenetic tree inferred using this dataset is presented in Figure 5. *P. vandenbrandei* has a very well-supported sister relation with *P. nainianus*. It has formed a clade with *P. minor* Sharma, Sharma & Khan, 1986; *P. nainianus*, *P. chongqinjensis, P. pedrami, P. baldaccii* Raski, 1975a, *P. leptos* Raski, 1975a and *P. rostrocaudatus* Huang & Raski, 1987, with maximal support (BPP = 1.00).

## Discussion

The objective of this study was the morphological and molecular characterization of the population of *Paratylenchus vandenbrandei* from Iran, which has not previously been documented in that country. The stylet length, number of lines in the lateral fields, and presence or absence of advulval flaps are considered to be robust characteristics for species identification of *Paratylenchus* (Ghaderi et al., 2014, 2016). Identification of *Paratylenchus* species based only on morphology and morphometric data is not always reliable, exposing a great phenotypic plasticity with inadequate species-specific diagnostic characters (Palomares-Rius et al., 2022). Consequently, *P. vandenbrandei* was studied using an integrative approach combining morphology, morphometry, and molecular data. Previous studies on this species have been based on traditional methods, but our present study defined the phylogenetic relationships of the species with related species for the first time.

In this present study based on 28S rDNA phylogeny, *P. vandenbrandei* clustered with *P. neonanus* and *P. nainianus*. Based on ITS rDNA phylogeny, the species has formed a clade with *P. minor*, *P. nainianus*, *P. chongqinjensis*, *P. pedrami*, *P. baldaccii*, *P. leptos*, and *P. rostrocaudatus*, but is clearly separate from these species in both phylogenies. According to a species grouping by Ghaderi et al. (2014, 2016), *P. chongqinjensis*, *P. leptos*, and *P. rostrocaudatus* are similar to the *P. vandenbrandei*, belonging to group two. *P. neonanus*, *P. nainianus*, *P. pedrami*, and *P. baldaccii* belong to group three, with four lines in the lateral fields, stylet length less than 40 μm, and advulval flaps present. *P. minor* belongs to group four, with no advulval flaps. Similar results have been achieved in some recent molecular phylogenies (Clavero-Camacho et al., 2021a, b; Palomares-Rius et al., 2022; Rosmaninho et al., 2022; Álvarez-Ortega et al., 2023).

Molecular markers can support identification of *Paratylenchus* species even when morphological characters might be variable and no overlapping ranges can be found (Clavero-Camacho et al., 2021a). In addition to morphological differences, *P. vandenbrandei* also differed from related species in molecular markers. Considering the various species of the genus *Paratylenchus* that have been reported from Iran, based on morphology and morphometry alone, it seems that more species of the genus will be found if molecular studies are carried out.
